# What is known about the role of rural-urban residency in relation to self-management in people affected by cancer who have completed primary treatment? A scoping review

**DOI:** 10.1007/s00520-020-05645-0

**Published:** 2020-08-03

**Authors:** David Nelson, Ian McGonagle, Christine Jackson, Ros Kane

**Affiliations:** School of Health and Social Care, University of Lincoln, Brayford Pool, Lincoln, LN6 7TS UK

**Keywords:** Cancer, Survivorship, Self-management, Urban, Rural

## Abstract

**Purpose:**

Despite wide acknowledgement of differences in levels of support and health outcomes between urban and rural areas, there is a lack of research that explicitly examines these differences in relation to self-management in people affected by cancer following treatment. This scoping review aimed to map the existing literature that examines self-management in people affected by cancer who were post-treatment from rural and urban areas.

**Methods:**

Arksey and O’Malley’s framework for conducting a scoping review was utilised. Keyword searches were performed in the following: Academic Search Complete, CINAHL, MEDLINE, PsycINFO, Scopus and Web of Science. Supplementary searching activities were also conducted.

**Results:**

A total of 438 articles were initially retrieved and 249 duplicates removed leaving 192 articles that were screened by title, abstract and full text. Nine met the eligibility criteria and were included in the review. They were published from 2011 to 2018 and conducted in the USA (*n* = 6), Australia (*n* = 2) and Canada (*n* = 1). None of the studies offered insight into self-managing cancer within a rural-urban context in the UK. Studies used qualitative (*n* = 4), mixed methods (*n* = 4) and quantitative designs (*n* = 1).

**Conclusion:**

If rural and urban populations define their health in different ways as some of the extant literature suggests, then efforts to support self-management in both populations will need to be better informed by robust evidence given the increasing focus on patient-centred care. It is important to consider if residency can be a predictor of as well as a barrier or facilitator to self-management.

## Introduction

### Purpose

The growing number of people living with and beyond cancer [1], in part due to advances in early detection, diagnosis and treatment, presents significant challenges for long-term care and the management of complications and side effects of treatment. Although research indicates that the majority of people affected by cancer are interested in managing their own health and healthcare [2, 3], reticence to engage in lifestyle and behavioural change, or uncertainty about how to do so, have also been reported [4].

Self-management is argued to be significant at all stages of the cancer journey, particularly in the post-treatment phase, as a result of reduced involvement with, and access to healthcare professionals [5], which leads to isolation and poor access to post-treatment support and resources. A need for further research to enhance understanding of experiences after treatment, and to support self-management, has been acknowledged [6].

Whilst there is no ‘gold standard’ definition of self-management [7], it has been described as ‘approaches used by the individual affected by cancer (or life limiting illness) and its effects to optimise living (with the illness and its effects)’ [8]. In the UK, the National Cancer Survivorship Initiative (NCSI) expanded this further and defined cancer self-management as awareness and active participation by the person affected by cancer in their recovery, recuperation and rehabilitation, to minimise the consequences of treatment, and promote survival, health and well-being [9].

Despite it being difficult to define and categorise, the concept of self-management has become well established within the cancer survivorship literature over the last decade [5, 10–22]. This has been bolstered by the shift in perception from cancer predominantly being an acute illness, to one that requires long-term management long after active treatment has ended.

Environmental factors (e.g. social and community support) have been recognised as key influencers of successful self-management [15]. However, there is no explicit reference in the literature to the role or influence of rural-urban residency.

It has been documented that social and community support [22] and access to health services can differ greatly depending on where an individual lives [14], but there is still no robust evidence that explicitly examines the role of rural-urban residency in relation to self-management among people affected by cancer who are post-treatment.

Both positives and negatives to rural and urban living have been identified [22, 23]. Research within the general population has highlighted the benefits of rural living and ‘green spaces’ in terms of improving physical and mental health [24, 25], and there are a range of characteristics belonging to rural communities that have the potential to benefit people affected by cancer [22]. For example, rural communities frequently value close relationships with family and friends, community members and religious institutions [26], which can all be significant sources of social support [22], vital to coping with or minimising emotional distress, when experiencing a traumatic life event such as a cancer diagnosis. Rural and urban areas can differ in the availability of social support delivered in the community [22]. Indeed, qualitative work identified the benefit of increased community support experienced by women affected by breast cancer who were living in rural compared with urban areas [23]. It has been argued that future interventions need to be designed to capitalise on the high levels of community trust in rural settings [27].

Rural residents tend to have higher cancer mortality than urban residents [28, 29], and people affected by cancer in rural areas are reported to face a range of additional challenges compared with their urban counterparts [30], including longer travel distances for treatment, limited access to medical care, support services and health and social care facilities [31–33]. Other factors include unmet psychosocial needs [30, 34] as well as increased risk for poorer health outcomes [35], poorer long-term survival [36] and a lack of relevant information [30]. Furthermore, attitudinal and structural barriers to help-seeking often differ between rural and urban populations [27, 30] which could consequently influence engagement with self-management.

This review therefore aims to investigate the phenomenon of self-management in people affected by cancer who were post-treatment, and to understand the role of rural-urban residency in relation to this.

The objectives were:

- To map the existing peer-reviewed academic literature examining self-management in people affected by cancer who were post-treatment from rural and urban areas.
- To determine the extent and type of evidence available.
- To identify any gaps in the evidence for further research.

## Methods

The methods for this review were based on Arksey and O’Malley’s [37] five step framework for scoping reviews: (1) identifying the review question; (2) identifying the relevant studies; (3) selecting the studies; (4) charting the data; and (5) collating, summarising and reporting the results. Each individual step is reported below. The adoption of this methodological framework ensured that the review process was methodical, rigorous and transparent [38].

### Identifying the review question

The question ‘What is known about the role of rural-urban residency in relation to self-management in people affected by cancer who have completed primary treatment?’ was developed to guide the search strategy.

### Identifying the relevant studies

Parameters for searching were decided at the outset. There were no limits placed on publication dates; however, only studies in English could be included due to lack of access to funds for translation. Inclusion and exclusion criteria (see Table 1) were devised at the outset and refined again following initial searching.

**Table 1:** Inclusion and exclusion criteria

Criterion	Inclusion	Exclusion
Time period	Any	-
Language	English only	Studies published in languages other than English.
Literature	Peer-reviewed academic literature	Non-peer-reviewed academic literature
Population	Adults who were 18 and over who had completed primary treatment for cancer.	Under 18; people currently undergoing active cancer treatment, people in receipt of palliative/end of life care. Studies exclusively on family members/carers/health and social care professionals
Study focus	Report information on the experiences of self-management in survivorship in relation to rural-urban geography.	There is no data directly in relation to self-management and the influence of rural-urban geography.
Study design	Quantitative, qualitative and mixed methods designs as well as relevant literature reviews.	-
Geographical location of study	Any	-

The final string that was used to search was as follows: (self-manage or ‘self-manage’ or self-management or ‘self-management’ AND cancer or neoplasms or oncology or tumour or tumor or malignancy AND surviv* or ‘living with cancer’ or ‘living with and beyond cancer’ or ‘affected by cancer’ AND rural or remote or isolated or regional or ‘small town’ or community or urban or cit*).

The databases searched were as follows: Academic Search Complete, CINAHL, MEDLINE, PsycINFO, Scopus and Web of Science. This ensured that a wide range of databases relating to nursing, health and social care, mental health and the behavioural sciences were included. A summary of the contents of each database can be found in Table 2. Searches were conducted on the following dates: 10/08/16; 01/08/17; 02/07/18; 23/07/19. The review was conducted as part of a doctoral thesis which warranted multiple search dates to ensure that the results were up to date prior to submission of the thesis in October 2019. Additionally, supplementary searching was performed on Google Scholar throughout the duration of this review. PROSPERO, the International Prospective Register for Systematic Reviews and the Cochrane Library were searched to ascertain if there were any similar literature or systematic reviews that were ongoing or completed. In this case, there were not.

**Table 2:** Overview of databases searched

Name of database	Contents	Platform/interface
Academic Search Complete	Multi-disciplinary journals, reports and proceedings.	EBSCO Host
CINAHL	Journals related to nursing and allied health issues.	EBSCO Host
MEDLINE	Journals related to life sciences, particularly biomedicine.	Ovid, EBSCO Host
PsycINFO	Peer-reviewed journals related to mental health and the behavioural sciences.	EBSCO Host
Scopus	Abstract and citation database of peer-reviewed research literature from scientific, technical, medical and social science fields and, more recently, also in the arts and humanities.	SciVerse
Web of Science	A multi-disciplinary database containing journals related to medical and social issues among others.	Thomson Reuters
PROSPERO	Protocol details for systematic reviews relevant to health and social care, welfare, public health, education, crime, justice and international development where there is a health-related outcome.	www.crd.york.ac.uk/prospero/
Cochrane	Database of systematic reviews.	www.cochranelibrary.com/
Google Scholar	Academic literature across a range of publishing formats and disciplines.	scholar.google.com

### Selecting the studies

**Fig. 1:**
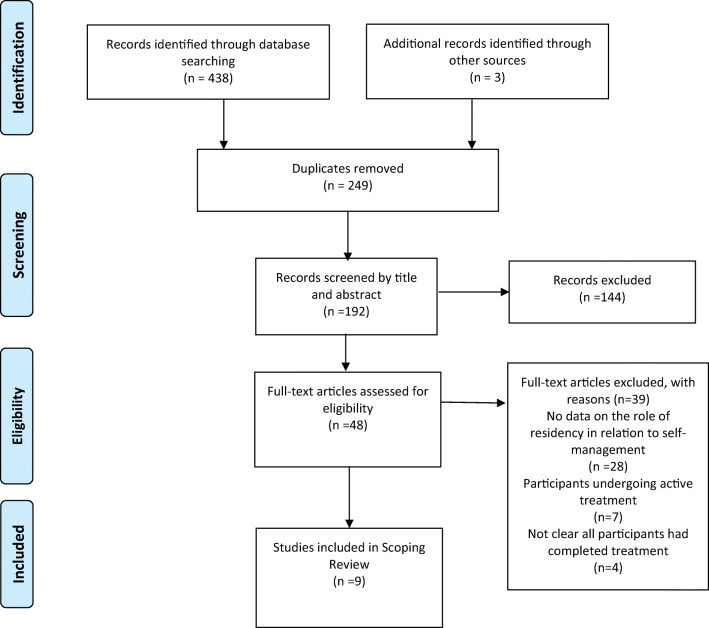
Flow diagram of scoping review

A total of 438 articles were initially retrieved across the six primary databases (Academic Search Complete, *n* = 89; CINAHL, *n* = 50; MEDLINE, *n* = 92; PsycINFO, *n* = 40; Scopus, *n* = 67; Web of Science, *n* = 100) and exported into the reference management software EndNote X8. A further three articles were retrieved from additional sources such as Google Scholar and reference lists from included articles. The duplicate articles (*n* = 249) were removed leaving 192 articles that were first screened against the inclusion/exclusion criteria by title. A total of 59 articles did not meet the inclusion/exclusion criteria after title screening. Next, the abstracts were read for the remaining 133 articles and they were again screened against the study eligibility criteria. A total of 48 were taken forward for full text screening. Thirty-nine articles were excluded following full-text screening meaning that nine were included in the final review and reported on in the results. The main reason for articles being excluded at full-text screening was due to not reporting any data that directly referred to the role of rural-urban residency on self-management (*n* = 28). Other reasons for exclusion were study participants undergoing active treatment (*n* = 7) or a lack of clarity about whether the participants had completed primary treatment (*n* = 4). Two reviewers (DN and RK) screened by title, abstract and full text and discrepancies around eligibility were reviewed by a third reviewer (IM) until agreement was reached. The search process is reported in Fig. 1.

### Charting the data

The next stage involved charting the data according to an analytical framework that facilitated sorting the material into a data extraction table [38] which can be found in Table 3. The table was created by the research team in order to meet the study objectives that were posited at the beginning of the review. Data was charted by one researcher (DN) and subsequently checked by another two members of the team (IM and RK). Standard information such as authors, year of publication, study setting, aim, methods, study population, findings in relation to self-management and residency were collected from all included articles.

**Table 3:** Data extraction table

Authors	Year	Setting	Aim	Methods	Participants	Findings in relation to self-management
Adams, N., Gisiger-Camata, S., Hardy, C. M., Thomas, T. F., Jukkala, A., & Meneses, K.	2017	Alabama, USA.	To better understand unique rural African American breast cancer survivor survivorship experiences and needs in the Alabama Black Belt.	Qualitative (focus groups/interviews) Survey used initially for demographic and treatment data.	African American breast cancer survivors (*n* = 15)	- Importance of social support from friends/family and healthcare providers. - Lack of survivorship education in the rural setting. - Participants identified needs for information about survivorship self-management, in particular around managing treatment-related side effects. - Spirituality and religion were prevalent coping strategies.
Gisiger-Camata, S., Adams, N., Nolan, T. S., & Meneses, K.	2016	Alabma, USA.	Community-based participatory research and multi-level assessment were used to (a) engage rural community leaders, survivors, and providers; (b) analyse and report results of discussion groups to understand survivorship concerns and preferences; (c) integrate discussion group findings to develop, tailor and deliver reach out; and (d) evaluate reach out with regard to satisfaction and helpfulness	Mixed methods 16 discussion groups with RBCS	Rural breast cancer survivors (*n* = 16)	- Self-management concerns: (1) fatigue, (2) pain, (3) depression, (4) lymphedema, (5) bone health osteoporosis, (6) hair loss, (7) sexual function, (8) hot flashes and menopausal symptoms and (9) comorbidities - Fears around being lost in transition, preferences around cancer support and concerns about cancer surveillance and health.
Glasser, M., Nielsen, K., Smith, S. N., & Gray, C.	2013	Illinois, USA.	The purpose of this study was to collect information to better understand the psychosocial needs of rural survivors of cancer and their significant others.	Quantitative (survey)	Rural cancer survivors (*n* = 29) and partners (*n* = 15)	- Over half at risk for depression and 34% reported some psychosocial problems —side effects or complications from treatment, emotional support or the impact of cancer on social relationships. - Those in rural areas need a team approach to meeting psychosocial needs.
Lally, R. M., Eisenhauer, C., Buckland, S., & Kupzyk, K.	2018	Nebraska, USA.	To obtain rural breast cancer survivors’ perceptions of the quality and usability of CaringGuidance™ After Breast Cancer Diagnosis, a web-based, psycho-educational, distress self-management program; and explore the feasibility of gathering survivors’ perceptions about CaringGuidance™ using online focus groups.	Primarily qualitative (online focus groups) Did collect some demographic and quant.	Rural breast cancer survivors (*n* = 23)	- Practical to recruit and retain rural people affected by cancer for research and testing of an intervention. - Rural participants willing to participate with digital technologies for self-management (emotional distress) - Challenges negative findings around rural internet use.
Lawler, S., Spathonis, K., Masters, J., Adams, J., & Eakin, E.	2011	Australia (range of locations classed as ‘rural’).	To explore and examine experiences and perceptions of follow-up care (medical and psychosocial) after active treatment for breast cancer among women living outside major Australian cities.	Qualitative – telephone interviews	Rural breast cancer survivors (*n* = 25)	- Limited access to medical follow-up care, psychosocial and lifestyle support programmes in rural settings. - Lack of community-based support programmes was a key concern. - Some participants given information about support that was not available in their area. - Desire for peer support – proactive in seeking this using telephone and the internet.
Loudon, A., Barnett, T., & Williams, A.	2017	Tasmania, Australia.	To describe the experiences of women taking part in a yoga intervention trial for breast cancer-related lymphoedema.	Qualitative - interviews	Rural breast cancer survivors (*n* = 15)	- Holistic practices like yoga can be successful in the rural setting. - Participants reported improved well-being, increased awareness of their body and improved physical, mental and social functioning. - Intervention also provided an opportunity to share experiences and for peer support.
McNulty, J. A., & Nail, L.	2015	Pacific Northwest, USA	To compare the impact of cancer in rural- and urban-dwelling adult cancer survivors living in 2 regions of the Pacific Northwest.	Mixed methods	Cancer survivors Quantitative (*n* = 132) Qualitative (*n* = 19)	- Significant differences between rural and urban respondents detected. - The interview highlighted further differences in relation to accessing healthcare, care co-ordination, connecting/community, thinking about death and dying, public/private journey and advocacy. - Rural participants tended to advocate for themselves, their diagnosis, survivorship and for improved healthcare in their communities.
Purtzer, M. A., & Hermansen-Kobulnicky, C. J.	2013	Wyoming, USA.	The study objective was to examine the meaning of self-monitoring practices within the context of rural patients’ responses to internal and external information.	Qualitative – semi-structured interviews	Cancer survivors (*n* = 20)	- Cognitive, affective, interpersonal, and symptomatic factors that informed self-monitoring which subsequently facilitated a sense of control and self-advocacy. - Cognitive – collected and critically appraised info they were given, note taking and recording information they were given were helpful. - Affective – fear anxiety and frustration but these negative feelings facilitated a desire to learn about cancer and manage it. - Interpersonal factors (informal and health professionals) vital to learning about cancer, treatment side effects and self-management in general. - Symptomatic – adverse treatment side effects. - Rural respondents reported ‘active’ rather than ‘passive’ coping strategies.
Stephen, J., Rojubally, A., Linden, W., Zhong, L., Mackenzie, G., Mahmoud, S., & Giese-Davis, J.	2017	British Columbia and Yukon, Canada.	The study aims were to examine proof of concept—feasibility, acceptability and usefulness—and to hone methods for a formal RCT.	Mixed methods Feasibility study with qual component	Breast cancer survivors (n = 105)	- Rural women benefited from online support group with psycho-education more so than those in urban areas. - The group supported self-management and facilitated focused and meaningful discussions that reduced illness-related stress.

### Collating, summarising and reporting the results

The final stage was to collate, summarise and report the results of the included studies. This was initially done by one researcher (DN) and then checked for accuracy by two members of the team (IM and RK). Scoping reviews aim to provide a descriptive account of the available research and do not normally attempt to appraise the literature utilising a quality assessment tool [38]. Therefore, a quality assessment was not conducted. An overview of the included studies is reported in the results below.

## Results

A total of nine articles were included in the review [39–47]. The studies were published from 2011 to 2018 and conducted in the USA (*n* = 6), Australia (*n* = 2) and Canada (*n* = 1). Out of the nine articles, four used qualitative methods, four used a mixed methods design and one was conducted using solely quantitative methods. Five of the studies were with people affected by breast cancer and the other four included participants who had been affected by a range of cancers.

A qualitative study with African American women from rural Alabama who had been affected by breast cancer (*n* = 15) [39] highlighted the need for social support from family and friends, as well as healthcare providers. The study highlighted a lack of survivorship education and support in their area. Furthermore, participants identified their needs for information about survivorship self-management, notably around managing treatment-related side effects. Within this context, spirituality and religion were crucial to coping with a cancer diagnosis and the effects of its treatment.

Further qualitative research [40] utilising semi-structured interviews with adults from a rural state in Western America who had completed treatment for cancer (*n* = 20) found four factors (cognitive, affective, interpersonal and symptomatic) that informed self-monitoring which subsequently facilitated a sense of control and self-advocacy. Self-monitoring can be considered a specific self-management strategy that involves patient awareness of thought processes, activities and physical symptoms in addition to the measuring, observing, recording and tracking of signs and symptoms. In terms of cognitive factors, participants collected and critically appraised the information that they were given. Some participants found note taking and recording information helpful to keeping track of and managing their situation. Information came from a range of sources such as health professionals, family, friends, support groups and the internet. Affective factors involved feelings of fear, anxiety, frustration, uncertainty and helplessness. However, these negative emotional responses facilitated a desire to learn about cancer and how best to manage it. Interpersonal relations were vital to learning about cancer, treatment side effects and self-management in general. For some, these were limited to interactions with only health professionals whereas for others, these extended to reliance on friends, family and support groups. Finally, the symptomatic factor refers to adverse treatment side effects, and participants would keep track and document these in the hope of minimising or managing them better in the future. For the most part, these rural participants reported ‘active’ coping strategies as opposed to ‘passive’ although there were no comparisons made to those from urban areas.

A recent feasibility study [41] highlighted that it is practical to recruit and retain people affected by breast cancer from rural areas for online focus groups and testing of a web-based education and self-management programme. The study is evidence that rural women are willing to participate in online focus groups and use web-based self-management support. Indeed, this was endorsed as an appropriate self-management tool for managing emotional distress, and [41] the authors maintain that knowing this is important to overcoming negative perceptions about rural internet use and this could be a suitable strategy to improve rural mental health disparities.

Further mixed methods research [42] with young women (under 50) who had a diagnosis of breast cancer (*n* = 105) where participants were randomised to two groups (online support group with psycho-education compared with self-help psycho-education), indicated that women who were from semi-rural and rural areas benefited from an online support group with psycho-education more so than those from urban areas. Moreover, the online support group that was professionally led supported self-management and facilitated focused and meaningful discussions that reduced illness-related stress. Notably, the study was also successful in outreach efforts to rural and semi-rural locations that normally lack psychosocial services and self-management support compared with their urban counterparts.

A qualitative study by [43] examined a yoga intervention as a tool to facilitate self-management with a sample of rural Australian women (*n* = 15) who had experienced lymphoedema as a consequence of treatment for breast cancer. The participants were highly motivated as evidenced by their high level of compliance regardless of having to travel for an hour and a half to attend the yoga sessions. However, the small sample size raises questions as to whether this would be replicable to a larger population. That said, holistic therapies such as yoga offer a range of practices that can be tailored according to the needs of the individual. Participants reported improved well-being and increased awareness of their body, as well as improved physical, mental and social functioning. The intervention also provided a place for them to share experiences with their peers, and the authors argue that yoga has the potential to augment and provide additional benefit to current self-management and treatment practices for women with breast cancer-related lymphedema.

Research into the delivery and development of the Reach Out to Rural Breast Cancer Survivors initiative that was delivered within a rural setting in four rural counties in Northeast Alabama in the USA [44] highlighted four major concerns through content analysis of discussions with sixteen women who were post-treatment. The first major concern was self-management in survivorship. The other three major concerns were fears around being lost in transition, preferences for support and concerns about cancer surveillance and health. The authors then identify a further nine themes from the data that they suggest related to self-management concerns: (1) fatigue, (2) pain, (3) depression, (4) lymphedema, (5) bone health osteoporosis, (6) hair loss, (7) sexual function, (8) hot flashes and menopausal symptoms and (9) comorbidities. These themes were then used to inform the content of the Reach Out intervention. For example, practical self-management tips on how to locate local resources were integrated into the programme, as well as specific tips to address sexuality and intimacy issues.

A pre-tested survey to ascertain general and mental health, quality of life and demographics with rural people who were personally diagnosed with cancer and was not undergoing any active treatment (*n* = 29) , and their partners (*n* = 15) [45] showed that over half of those with a personal history of cancer were at risk for depression and 34% reported some type of psychosocial problem that required assistance, such as management of treatment-related side effects or complications of treatment, emotional support or the impact of cancer on social relationships. Although these findings should be interpreted with caution given the small sample size, the authors suggest that those in rural areas are likely to require a team approach to meeting psychosocial needs.

A qualitative study (*n* = 25) on experiences of follow-up care (medical and psychosocial) following breast cancer treatment for women living outside major Australian cities [46] highlighted that there was limited access to medical follow-up care, as well as psychosocial and lifestyle support programmes in rural settings. Interestingly, lack of community-based support programmes was a key concern, and some participants were given information about support that was not available in their areas. Several participants wanted peer support with other women affected by cancer; some were proactive in sourcing this in the local area, using the telephone and internet to access this. Furthermore, there should be greater co-ordination of care between health professionals to improve communication and reduce the burden on both, the patient and the medical system.

Finally, a mixed methods study compared the impact of cancer in rural and urban-dwelling adults in two regions of the Pacific Northwest [47] using a questionnaire (*n* = 132), and in-depth interviews (*n* = 19) showed statistically significant differences between rural and urban when it comes to differences in body concerns, worry, negative impact and employment concerns. The interview data indicated further differences in relation to accessing healthcare, care co-ordination, community, thinking about death and dying, public/private journey and advocacy. Rural participants tended to advocate for themselves, their diagnosis, survivorship and for improved healthcare in their communities. The advocacy emerged as seeking a second opinion, accessing support resources, asking questions and seeking answers and fighting for their financial and employment rights. The rural participants in particular engaged with community advocacy by fundraising, volunteering with survivorship organisations and speaking publicly about survivorship issues.

## Discussion

Firstly, none of the studies that met the eligibility criteria offered direct insight into self-managing cancer within a rural-urban context in the UK. Similar to much of the broader literature on survivorship and geography [30, 35, 48–50], the included studies were conducted in the USA, Canada and Australia. Whilst the studies that were included in this review shed light on some of the self-management experiences within a rural-urban setting, they are from countries with different healthcare systems and services in comparison with the UK, thus warranting further investigation in a UK setting. Despite the increasing body of work that explicitly focuses on self-management and cancer within a UK context [13, 17, 18, 51–54], there are no studies that have reported data on rural-urban residency in relation to this. Equally, research comparing outcomes between rural and urban people affected by cancer has tended to focus on the post-diagnosis stages, as well as survival rates, and has yet to examine the influence of residency on longer-term survivorship outcomes, such as self-management post-treatment.

Interestingly, eight of the nine articles used some form of qualitative methods, either in isolation or in combination with quantitative methods as a mixed methods design. Qualitative methods seem an appropriate choice to shed light on and explore the in-depth experiences of people affected by cancer; however, there is a considerable need for larger studies with increased sample sizes that utilise quantitative and mixed methods designs. Particularly, where geography is concerned, there is a need for research with a range of cancer types over several different regions so the results can be generalisable to wider rural and urban populations. Much of the research that was included focused on one specific location, with relatively small sample sizes, and in some cases with the same cancer type such as breast (*n* = 5) which limits the extent to which the findings can be inferred to other settings and population groups.

Only two of the included studies collected data from participants in urban areas, as well as rural [42, 47]. Whilst these two studies identified some of the similarities and differences with rural and urban populations in relation to ‘self-management’, they do not explicitly focus on ‘self-management’ as one of the primary variables under investigation. Indeed, self-management studies that compare between rural and urban with people affected by cancer are non-existent within the UK and international literature. With that in mind, researchers in psychosocial oncology should be encouraged to collect both quantitative and qualitative data on rural-urban residency to enhance their analyses. Whilst the studies in this review focus directly on rurality signpost to the perceived differences with rural and urban living when presenting their background and context, they do not collect and analyse data from urban populations in their own study which limits the extent to which we can consider these findings unique to the rural setting without a comparator group. Some of the existing American cancer research that has compared between the two on mental health [55], health status and health behaviours [35, 50] have used official statistics to categorise and define rural-urban residence, and other researchers, where appropriate, should be supported to do the same. In fact, utilising the same methods for defining and measuring rural-urban status would support comparison between researchers, at the very least, on a national and regional level and promote wider collaboration in the field. Furthermore, interventions need to account for geography and the specific traits of rural and urban populations; therefore, cancer survivorship scholars should be encouraged to take note of this when designing and implementing interventions.

With regard to the findings from the studies themselves, they identified the salient needs of those from rural areas and that emotional management seems to be a significant concern [45]. This is not surprising given that geographic and emotional isolation is often associated with rural living as seen in some of the wider literature [56, 57]. However, in contrast, it is argued that survivorship experiences are similar regardless of rural-urban residence with the exception of access to specialised survivorship services and resources being the primary difference, as well as a considerable challenge for those in remote locations [44]. A potential solution could be the use of IT and as digital technologies and e-health applications have the potential to support and address needs with rural populations who have been affected by cancer [46]. That said, for this to work, it would be dependent on internet access which can still be limited (or even non-existent) in very rural and remote locations. Indeed, recent work in the UK [58] with people affected by breast cancer suggests that an e-health app could be successful in facilitating peer support and coping strategies. At the same time, it has been shown that social networking does not always provide added benefit, and consideration needs to be given to what stage of the cancer journey this is delivered to the individual [4]. With that in mind, if future time and financial resources are to be invested in the design and utilisation of digital technologies to support health behaviours and self-management, academics and health professionals have a duty to ensure that these are designed and tailored to the needs of both rural and urban populations. Whilst this review was conducted prior to the global pandemic, COVID-19, some people affected by cancer might now feel particularly vulnerable, especially when confined to their homes as a consequence of cancer or other comorbid conditions. It is therefore likely that demand for digital support will increase regardless of geography, where people can learn from professionals and peers without the need for face-to-face contact.

In one study exploring the differences and similarities between rural and urban living [47], the findings challenge the widespread assumption about the perceived negative impact of rural living on health outcomes. Notably, some of the wider literature reinforces a range of characteristics belonging to rural communities that have the potential to benefit people affected by cancer [22, 23]. Perhaps, not surprisingly, a common theme in the literature is ‘community’ and research shows that access to healthcare might not be the most salient concern when it comes to the survivorship experience. Social and community support has specifically been accounted for in a framework for recovery of health and well-being in cancer survivorship [15], and it has been posited that community support can differ depending on where an individual lives [22]. This study sheds light on the role of empowerment in rural communities and future researchers in the field should take note. However, the majority of their sample were female and had been affected by a breast cancer diagnosis where there might be more resources available to support recovery and self-management. Additionally, females tend to be more socially active and to seek other forms of social and emotional support compared with males. Nonetheless, the findings highlight some interesting traits of rural communities in relation to cancer survivorship that warrants further data collection with more diverse samples.

## Limitations

Whilst the methods and results from the scoping review identified a gap in the extant literature, notably, that no studies offered direct insight into self-managing cancer within a rural-urban context in the UK, the databases chosen ensured that a wide range of literature in relation to nursing, health and social care, mental health and the behavioural sciences was searched. However, the review was not without its limitations and the search strategy could have been extended to include additional databases such as the Rural and Remote Health Database via Informit Online, although the host institution did not have access at the time of the research. The grey literature was not searched as one of the primary objectives was to map the peer-reviewed academic research in this area. Although to gain a deeper understanding of the field, future reviews should consider exploring the subject-specific grey literature. Given that this was not a full systematic review, the review protocol was not registered; however, this could have allowed for initial peer review of proposed methods and increased transparency and awareness of the research. Finally, the search terms could have been extended to include related terms such as ‘self care’, ‘self help’, ‘self education’ and ‘patient education’, thus ensuring a more comprehensive and thorough search of the academic literature.

## Conclusion

Given that a scoping review is not meant to be exhaustive but serves to offer the reader with a good sense of the literature, it is possible that some relevant publications were not included. Regardless, the number of included articles (*n* = 9) serves to illustrate that this is an under-researched area, particularly with UK populations, who have completed primary cancer treatment. To date, there is no existing research that examines and compares self-management with people affected by cancer who have completed treatment in rural and urban parts of the UK.

If rural and non-rural populations define their health in different ways as some of the literature suggests, then efforts to support self-management in both populations will need to be better informed by robust evidence given the increasing focus on patient-centred care [59]. It is therefore important to consider if residency can be a predictor of self-management, as well as what acts as a barrier and/or facilitator to self-management; the findings can then be used to inform support that is delivered to people affected by cancer and ensure that it is tailored to population needs in line with geography. Future studies with people affected by cancer should consider collecting data on rural-urban residence where appropriate. This can then be utilised to inform interventions and support based on the needs of both rural and urban populations.
